# Correcting misinformation by health organizations during measles outbreaks: A controlled experiment

**DOI:** 10.1371/journal.pone.0209505

**Published:** 2018-12-19

**Authors:** Anat Gesser-Edelsburg, Alon Diamant, Rana Hijazi, Gustavo S. Mesch

**Affiliations:** 1 The Health and Risk Communication Research Center, University of Haifa, Haifa, Israel; 2 School of Public Health, University of Haifa, Haifa, Israel; 3 Department of Sociology, University of Haifa, Haifa, Israel; University of Campania, ITALY

## Abstract

**Background:**

During epidemic crises, some of the information the public receives on social media is misinformation. Health organizations are required to respond and correct the information to gain the public’s trust and influence it to follow the recommended instructions.

**Objectives:**

(1) To examine ways for health organizations to correct misinformation concerning the measles vaccination on social networks for two groups: pro-vaccination and hesitant; (2) To examine the types of reactions of two subgroups (pro-vaccination, hesitant) to misinformation correction; and (3) To examine the effect of misinformation correction on these two subgroups regarding reliability, satisfaction, self-efficacy and intentions.

**Methods:**

A controlled experiment with participants divided randomly into two conditions. In both experiment conditions a dilemma was presented as to sending a child to kindergarten, followed by an identical Facebook post voicing the children mothers’ concerns. In the third stage the correction by the health organization is presented differently in two conditions: Condition 1 –common information correction, and Condition 2 –recommended (theory-based) information correction, mainly communicating information transparently and addressing the public’s concerns. The study included (n = 243) graduate students from the Faculty of Social Welfare and Health Sciences at Haifa University.

**Results:**

A statistically significant difference was found in the reliability level attributed to information correction by the Health Ministry between the Control condition and Experimental condition (sig<0.001), with the average reliability level of the subjects in Condition 2 (M = 5.68) being considerably higher than the average reliability level of subjects in Condition 1 (4.64). A significant difference was found between Condition 1 and Condition 2 (sig<0.001), with the average satisfaction from the Health Ministry’s response of Condition 2 subjects (M = 5.75) being significantly higher than the average satisfaction level of Condition 1 subjects (4.66). Similarly, when we tested the pro and hesitant groups separately, we found that both preferred the response presented in Condition 2.

**Conclusion:**

It is very important for the organizations to correct misinformation transparently, and to address the emotional aspects for both the pro-vaccination and the hesitant groups. The pro-vaccination group is not a captive audience, and it too requires a full response that addresses the public's fears and concerns.

## Introduction

### The challenge of social media for health organizations

The public sphere in the 21^st^ century has undergone a transformation generated by the adoption of online communication technologies. New media has become an important source of health information and a platform for discussing personal experiences, opinions and concerns regarding health, illnesses and treatment, and has contributed to the shift in the role of the public from passive recipients of information to an active and vocal entity [1, 2].

In the new media, during epidemic crises (whether familiar or new), the public receives information which is sometimes misinformation (deficient information) or disinformation (intentionally false information). This has implications for health-related decision making and public health behavior.

Today, as Samarajiva and Gunawardene [3] argue, because of the proliferation of new media platforms, the controlled dissemination of information is no longer an option for any government or health authority. In the 2015 measles outbreak in California, the story broke by way of a post tweeted by a pediatrician from San Diego, who wrote about the cases diagnosed and their connection to the Disney parks. The formal statement by the California Department of Public Health was issued only more than 15 hours later [4].

Health organizations are required to respond and correct information to gain the public’s trust and influence it to follow the recommended instructions. Research indicates that innacurate and misinformation provided to the public can trigger skepticism and resistance [5–7]. Furthermore, it can cause a boomerang effect, a reaction that is the opposite of the intended response to persuasion messages [8, 9]. Furthermore, quite often during epdemic crises health organizations are required to manage and communicate information to the public in conditions of uncertianty.

These major issues of correcting misinformation are currently of primary concern in the field of health communication, however there are still insufficient empirical studies in the field to help create a theoretical infrastructure alligned with the public sphere in the age of new media.

Many studies have indicated that in emergency and crisis situations the public seeks information in order to reduce uncertainty, and will use whatever means available to obtain it [10, 11]. In the event of an epidemic, people want to know the risk of infection (propability), how sick they could become if infected (severity), and what can be done to prevent and treat the disease. In order to be able to make decisions about using vaccines and drugs, they need information that will enable them to assess the risks and benefits involved [12]. Studies have also indicated the important role of healthcare workers, mainly pediatricians who work in primary health care, as source of information on vaccination [13–18].

The issue of vaccination is one of the major health sources of concern raised in social media. For example, during the 2009 H1N1 outbreak in the USA, there was a pronounced rise in the use of social media to express concerns related to the vaccine’s side effects and risks [19]. This discourse of doubts and concerns, as well as the anti-vaccination messages in the social media [20], may influence vaccination decisions and increase vaccination hesitancy and refusal trends among parents, which have been on the rise in recent years [21], with a significant contribution to suboptimal vaccination coverage with the resurgence of vaccine-preventable diseases [22–25].

### The increasing use of social media by health organizations

The Zika virus, Ebola, H1N1, MERS, polio and SARS outbreaks, as well as the HPV and measles vaccines, have stood at the center of medical and social controversies in recent years. Time and again, in crisis situations caused by disease outbreaks, the importance of health communication in new media has emerged. To help assess anxiety and concerns, scholars have suggested using web-based data analysis to gather insights into public responses to infectious disease outbreaks [19, 26]. The use of social media platforms has contributed to a radical transformation in the relationship between government organizations and the public. Social media has changed from a monologue to a dialogue, where anyone with information and communications technology (ICT) access can be a content creator and communicator [27].

Over the past decade, leading international health organizations and local governments have invested financial and human resources in order to mitigate the gaps in social media that were conspicuous in previous health crises between the organizations and the public, offering greater presence in social media. New guidelines, such as the Social Media Metrics for Federal Agencies [28], based on the White House's Digital Government Strategy [29], outline specific steps for governmental agencies to make digital information more "customer-centric." During the Ebola (2014) or the Zika (2016) outbreaks, social media enabled the CDC to be part of the sphere of active communication: to ask questions and get quick answers. By doing so, the CDC contributed to the public’s health literacy and uncertainty mitigation [30–32].

### Difficulties in using "social" (two-way communication) in social media in organizations

The theory of effective health communication of social media is based on the convergence communication approach [33]. In this approach, the information circle (providing and receiving information) between the organization and the public goes back and forth, until they converge on common ground. Fischhoff expressed this articulately: “The transition from a mechanistic and linear model of risk communication to a more participative and iterative approach can empower the public in the process of decision making about risk and uncertainty” [34].

Despite this impressive transformation, the use of social media by organizations is still in its infancy. While the literature indicates that health organizations on the international, national and local levels actively use social media, it also shows that this use is still very limited, as these tools serve primarily for mass information dissemination (similar to the traditional mass media), instead of as two-way dialogue mechanisms [26, 35].

### Adapting message to three subgroups: pro, hesitant and anti-vaccination

The importance of tailoring and adapting information to population subgroups is crucial in regards to epidemics and the need for vaccination [36, 37]. The pro-vaccination group (the group that vaccinates according to the health organizations’ recommendations) is still the largest group in the population. However, in recent decades there is a growing group called “hesitant parents,” which chooses whether to use vaccinations, and their timing, selectively. There is also a very small group of anti-vaccine parents who do not vaccinate their children at all for ideological reasons. Health organizations understand the importance of these two groups and are concerned about their growth, as they haven’t yet study and identified the proper strategies to reach them[38]. One of the challenges facing the health organizations in managing the two-way communication channels in a web-based social sphere is correcting or clarifying partial (mis-) or mistaken (dis-) information, to three different groups on social media (pro, hesitant and anti-vaccination). In a world containing partial, popular, missing or wrong information online and on social media, the health systems strive to communicate credible information directly from the healthcare organizations.

### Misinformation on social media

“Online news websites, forums, blogs, and Facebook posts create a unique blend of information sources, including scientific literature, medical professionals, and government representatives, as well as pseudoscientific research” [39]. Studies indicate that factors that increase the efficiency of misinformation correction concern warnings at the time of the initial exposure to this information, and recurring repetition of the correction by the organizations [40]. Bode and Vraga (2017) suggest that "Correction can work, when it is done quickly and clearly, and provides supporting evidence either through a related stories algorithm or a link offered by a social contact. In a world of ever-faster information diffusion, this has major implications for the actions organizations and individuals take to reduce health misinformation online" [41].

In the summer of 2016 the WHO declared the Zika virus a global threat. Avery [42] tried to examine the importance of monitoring the discourse on social networks by the organizations during the Zika virus outbreak and the effectiveness of that monitoring during crisis management. The results indicated that there was a positive correlation between monitoring the social networks and satisfaction of health crisis managers with the management of the Zika virus; crisis managers who examined the information conveyed on the networks could characterize and identify misinformation and concentrate on the relevant information.

In many cases, misinformation on social media is the result of rumors as in the case of the death of a 12-year-old Waukesha girl [43], deliberate fear-mongering as in the case of fatwas in Pakistan against polio vaccination, claiming that OPV sterilizes children [44] and the absence of full public communication by health organizations of the information they possess to the public. It is important to note that alongside deliberate disinformation by stakeholders, most of the discourse on social media stems from people’s desire to obtain additional information, which is sometimes not fully conveyed by the health organizations [1]. For example, in 2003 the Japanese Ministry of Health avoided publishing WHO data according to which some people in Japan had SARS symptoms. Furthermore, the ministry failed to initiate a prevention strategy in various communities in Japan, causing the disease to spread, because local hospitals did not prepare accordingly [45].

### Common correction by health organization: Is framing social media information as myth an effective strategy?

The most common way health organizations approach the correction of social media information is by calling it “myths.” This is as opposed to the information originating in the organizations, which is called “facts” [46]. Use of this strategy is common on a range of health issues [47–49]. This form of correction was found ineffective for two reasons. One, citing the information on the website as myth makes people remember the information even though it is untrue, or only partially true. For instance, a study of the American Diabetes Association (ADA) found that exposure to “myths” misled respondents and reduced their knowledge of the subject, while respondents who had not been exposed to myths showed more knowledge [50]. The second reason is that the public refused to accept a judgmental approach from the organization without scientific evidence. In two studies on public attitudes towards the MMR vaccine and the seasonal flu vaccine [51, 52], parents were presented with pro-vaccine information from the CDC website. However, these pro-vaccine messages failed to improve parents' attitudes toward vaccination. In fact, these studies reported a “backfire effect:” vaccine skeptics formed even stronger negative opinions about vaccinations after being given information intended to undermine the supposed connection between vaccinations and autism.

### Recommended (theory-based) effective risk communication: An integrative decision-making process

The principles of effective risk communication indicate the importance of transparent information from health organizations as they address the public’s concerns and worries [8]. Health communication literature and research on health-related decision making indicates that at times of crisis, people, including experts, make decisions based on a mixture of feelings, experience and analytic considerations [53–55]. The literature indicates that health organizations all over the world tend to ignore the emotional element, and by doing so miss out on leading an effective dialogue with the public. They also neglect to give the public the tools to read and understand information that can empower and increase perceived self-efficacy–as individuals and as communities[56].

People often tend to reject information corrections that contradict their attitudes[57], share content that is consistent with their own narratives while ignoring the rest[58], and allow the mistaken information to continue to effect their conclusions even when they bluntly admit the information is incorrect [59]. Nonetheless, other studies indicate that the correction of misinformation by the organizations has a positive effect on the public’s decision making [60, 61]. To continue examining crucial issues such as correcting misinformation, according to Nyhan, “a more comprehensive strategy is likely to be required" [51].

### Measles outbreaks in the world and in Israel

According to CDC data, between January 1 and July 14, 2018, 107 cases of the measles were reported in the U.S. in 21 different states and the District of Colombia. A record number of cases of measles were reported in the US in 2014: 667 cases in 27 states, the highest number reported since 2000 [62]. The next measles outbreak was detected in 2015 at a California amusement park, leading to a debate over the reasons for the contagion. Measles outbreaks also occurred in a number of European countries. Between September 1, 2017 and August 31, 2018, 13,547 cases of measles were reported to the European Surveillance System by 30 countries [63]. The official ECDC interpretation is that the main reason for the measles outbreak in European countries was suboptimal vaccination coverage [63, 64]. In Italy, which is among the countries with the highest percentage of cases reported between September 1, 2017 and August 31, 2018, the main factors underlying the resurgence of measles cases have been addressed in several studies [65–68]. According to Biellik et al. [69] in order to expedite and finalize measles and rubella elimination In the European region, "Genuine political commitment, increased technical capacity, and greater public awareness are urgently needed, especially in Western Europe".

In 2014 the World Health Organization defined Israel as a country where the measles had been eliminated, with a small number of reported cases and with no evidence of continued widespread contagion. But in 2016 the Ministry of Health received reports of eight measles patients, and by the end of 2017 it received 42 reports of cases of measles [70]. From January to March 2017 no cases of measles were reported in Israel at all, but in March a measles outbreak began, and 130 cases were detected in Israel. In response to the outbreak, the Ministry of Health issued a memo in April 2018 to increase awareness and refresh procedures in the health bureaus. Physicians were asked to report to the local health bureau every suspected case of measles, and district physicians were required to vaccinate every possible patient within 72 hours of exposure [71].

The measles outbreak in Israel generated discussion on the social networks. Parents, healthcare experts and health organizations issued posts and reactions about the measles and vaccination, which generated debates, especially between the pro-vaccination and hesitant groups [72]. The narrative centered mainly on the importance of vaccinating children to prevent contagion, the severity of the illness, who was at fault for the measles outbreak, and attitudes towards parents who do not vaccinate their children. In light of the debate and the misinformation that arose from the conversation on the social networks, and in light of the fact that there is very little empirical research in the professional literature on how health organizations should react and correct misinformation [73], this study wishes to fill that void by examining ways for health organizations to correct misinformation concerning the measles vaccination on social networks for two groups (pro and hesitant), having two specific objectives (1) To examine the types of reactions of two subgroups (pro, hesitant) to misinformation correction; (2) To examine the effect of misinformation correction on these two subgroups regarding reliability, satisfaction, self-efficacy and intentions.

## Materials and methods

### Experiment design

The research used mixed methods [74], with a main quantitative research (controlled experiment), along with qualitative research (open questions). This allowed us to go beyond assessing attitudes and perceptions through a survey and to use simulations in order to identify effective corrections tailored to different subpopulations.

### Variables

#### Control variables

Socio-demographic characteristics (sex, age, education, ethnicity, profession, marital status), and parental variables characteristics (number of children, children’s age).

#### Manipulated variables

Communication correction of misinformation regarding measles vaccination (common vs. recommended theory-based information correction).

#### Measured variables

This experiment included a number of simulations that required stimulation and realistic reaction time by the respondents. Therefore, we could not create indexes of questions for each variable, mainly because of the length of the experiment and time limits, so single questions were chosen. The variables that were tested are: Self-efficacy, reliability, satisfaction, information-searching, behavioral intentions. The variables were tested using the following questions: (1) Trust: Do you find the Health Ministry’s response credible? (on a seven-point response scale whereas very much so = 1 to very little = 7). The scale was reversed in the questionnaire and returned in the analysis; (2) Satisfaction: Are you satisfied by the response he received from the Health Ministry? (on a seven-point response scale whereas very satisfied = 1 to not satisfied at all = 7). The scale was reversed in the questionnaire and returned in the analysis; (3) Self-efficacy: Do you feel you have the tools to make decisions about sending your child to kindergarten? (on a seven-point response scale whereas very little = 1 to very much = 7). This appeared both after simulation 1, story 2 (the post) and after the response correction (the Health Ministry); (4) Information searching: After the Health Ministry’s response will you continue to search for information? (response scale: yes/no); (5) Behavioral intention whether to send child to kindergarten: After reading the Health Ministry’s response (correction), would you send your son/daughter to kindergarten? (response scale: yes/no); (6) Behavioral intention about vaccinating child: Following the Health Ministry’s response (correction), would you vaccinate your son/daughter? (response scale: yes/no).

### Assumptions and hypothesis

The assumption of this research is that theory-based misinformation correction will lead to a rise in satisfaction, trust, self-efficacy, and openness to the message. The opposite will cause a decline.

First hypothesis: An association will be found between the type of the Health Ministry’s information correction (common and recommended information corrections) and the two subgroups’ (pro, hesitant) levels of satisfaction, such that the recommended information correction will yield the highest satisfaction level among all groups.

Second hypothesis: A positive association will be found between the level of satisfaction with the information correction, the level of trust, and the level of self-efficacy among all groups, following the Health Ministry’s recommended (theory-based) misinformation correction.

### The research population

The experiment included 243 students, 65% of which work in health professions. Of them, 174 were students at the School of Public Health and 69 were in different departments of the Faculty of Social Welfare and Health at Haifa University in the years 2017–2018.

### Data collection procedure

The 174 students at the School of Public Health did the experiment using printed questionnaires in the classroom, with no dropouts (i.e. 100% response rate). The additional 69 students were an introduction to psychology course students from different departments of the Faculty of Social Welfare and Health who participated in an online version of the experiment, having identical questionnaires as in the printed version of the experiment. They were approached by emails enabling them to join the online experiment through a link to a Qualtrics software that was sent to them. 69 out of 85 students filled out the online questionnaire in full. Incomplete questionnaires were removed from the data on base of a small size effect (being only 6% out of the total questionnaires). The online experiment response rate was 21%. However, these students who participated in the online version of the experiment, were given the option to choose to participant in serval experiments simultaneously as part of their course requirements during a semester, therefore this response rate should not be considered as low in this unique setting. Randomisation within the students at the School of Public Health was achieved by administrating to questionnaires after they were pre-mixed (Condition 1 and Condition 2) blindly and re-grouped into one group. As per the other participants, randomisation was achieved by the Qualtrics software.

The rationale for selecting this population was twofold: (1) Because this was not a short length survey that could have been given to any population, but a research model that requires the respondents to undergo several simulations that takes 40–45 minutes, we used students who are able to make the time and effort; (2) Most of the students who participated in the experiment were public health professionals, most of whom work in the Israeli health system. We were interested in finding out how these respondents would react to two forms of correction by the Ministry of Health. We assumed that if they will respond positively to Condition 2, it would strengthen the research hypothesis.

Of the 243 participants, 126 received and answered experiment questionnaire version 1, and the remaining 117 answered experiment version 2 (participants with identical profiles). Of the subjects who were healthcare workers, the most common profession was nurse (31%)). Other professions included dietitians (19.5%), doctors (2%), occupational therapists (3.1%) and more. 136 participants were pro-vaccination, 72 were hesitant and 9 were anti-vaccination, according to their self-reports. In order to be considered pro-vaccination it was necessary to answer “yes” to the question "do you give your children all of the vaccinations according to the vaccination routine," and “no” to the question "are you selective in vaccinating your children." In order to be considered hesitant it was necessary to answer “yes” to the question "are you selective in vaccinating your children." In order to be considered anti-vaccination it was necessary to answer “no” to the question "do you give your children all of the vaccinations according to the vaccination routine," and “no” to the question "are you selective in vaccinating your children."

### The misinformation experiment

Participants were divided randomly (before we explained about the experiment) into two conditions: Condition A–Common information correction, and Condition B–Recommended information correction. The two groups in conditions A and B were presented with several simulations, after each one of which participants were asked to answer the recurring questions (see below). The two first simulations were identical for the two conditions. The first simulation presented a dilemma of parents whether to send their children to kindergarten during a measles outbreak, knowing that some of the kindergarten children were not vaccinated because of their parents' objection. In the next simulation they were shown a post by the mother of one of the kindergarten children, containing misinformation about measles and the ways it is contracted. In the following simulation, experiment participants in Condition A were shown a response by the official health authority (such as the Ministry of Health), trying to correct the misinformation in the mother's post. The correction was formulated as a common statement comprised of a short brief, an unequivocal message, and without addressing the emotional element (empathy, referring to fears and concerns) of the mother who wrote the post and other parents. Meanwhile, participants in Condition B were shown a recommended correction, providing full transparency about information on the disease and how it is contracted, addressing the emotional element (empathy, referring to fears and concerns) of the mother who wrote the post and other parents.

#### The misinformation experiment stages and questionnaire

**Stage 1—Presenting the dilemma:** Please read the following story and answer the questions; You are a mother or father who wants to send their son or daughter to kindergarten. The kindergarten is considered to be a very good one, is close to home and on the way to your work. It has a great teacher and a waiting list. The kindergarten has children who are not vaccinated for measles because their parents refuse to vaccinate them. The identity of the unvaccinated children is unknown. The teacher informed the parents that there are three children who are not vaccinated for measles and she is ethically precluded from telling them who they are.

**Stage 2—Facebook post:** Hi. I’m the mother of a four-year-old girl, Gili. I don’t usually post about these things, but I have to respond this time. I don’t understand why all this pressure from the Health Ministry and parents about the measles vaccination. My mother, may she be healthy, and her mother, who is about to turn 90, may she be healthy too, never got the measles vaccine, because it used to be considered a mild childhood disease that the body could deal with by itself and overcome. Children who get the measles manufacture antibodies and their immune systems are stronger. I read in a few places that children who are not vaccinated put vaccinated children at risk. What kind of nonsense is that? To the contrary, the vaccinated children who have the inactivated vaccine in their bodies can actually transmit the disease to the nonvaccinated children. I’m sick and tired of all these attempts to force parents to do things that are not necessary. I am a caring mother and that is why it is important for me to share this with you, dear mamas.”

**Same questions after stages 1 and 2:** (1) Following the story, would you send your son/daughter to the kindergarten? (Yes/No). Explain your answer; (2) Following the story, would you vaccinate your son/daughter? If you did not vaccinate at all (note: this question is for those who have not vaccinated their children at all), explain your answer. Will you continue to vaccinate your son/daughter with the second dose if you have vaccinated him/her with the first dose? Explain your answer; (3) After reading the story of the parents who do not vaccinate their children, to what extend do you identify with them? Please make a circle around the aspect that best describes your feelings on a scale of 1 (identify very much) to 7 (identify very little); (4) What do you think about parents who do not vaccinate their children? Do you think these parents are right? Please make a circle around the aspect that best describes your position on a scale of 1 (very much) to 7 (very little) after every option; (5) Do you feel you have the tools to make a decision about sending your child to the kindergarten?; (6) What information would you want to know about the vaccine in order to make the decision of whether to send your son/daughter to the kindergarten?; (7) Who will you turn to for information about the measles vaccine considering the dilemma presented in the story? Please mark the main two sources you would turn to (list of sources); and (8) Who do you think is the most reliable source for receiving the information? (Please mark a single answer).

**Stage 3—The Ministry of Health’s response to the post: Version 1**: “We would like to respond to the post that we received from one mother's social networks. Unfortunately, there are many parents like this mother who are not professionals. This mother is not a physician and therefore she cannot be trusted. We reiterate that it is necessary to vaccinate with the MMR vaccination against measles. Anyone who does not vaccinate is thereby risking their lives and the lives of those surrounding them. It is very important for all the children in the kindergarten to be vaccinated. Millions of people around the world die every year as the result of contraction and complications of the measles. Therefore, we call on all parents to vaccinate your children because vaccination saves lives!”

**Version 2**: “Hello, we would like to respond to the post of a mother that we received from the social networks. We respect the mother very much and understand her concerns following the outbreak of the measles and the position she presented in her post. Honestly, the arguments she so skillfully raised in her post seem to be reasonable. We would like to clarify scientifically some of the facts for all parents who are dealing with the same question: Measles is a common and contagious infectious disease transmitted through the respiratory system. The virus’s incubation period is between 10–14 days. The first typical symptoms of the disease include fever, runny nose, cough and conjunctivitis, which are not specific to measles. 3–4 days after the fever a typical measles rash appears. Over the years there have been numerous reports of serious complications including pneumonia and meningitis. It is important to emphasize that the immune system of vaccinated children is not weaker than that of children who have contracted measles, to the contrary. Vaccinated children have stronger immune systems that help them fight the virus when they are infected. Vaccinated children cannot transmit and infect other children because the vaccine that is inserted through the vaccination does not cause the disease. The effectiveness of two doses of vaccine ranges from 93–99%.”

**Questions after stage 3:** (1) After you read the Health Ministry's response will you send your son/daughter to the kindergarten? (Yes/No). Explain your answer; (2) Following the above reaction of the Health Ministry, will you vaccinate your son/daughter? If you did not vaccinate at all (this question is for those who have not vaccinated their children at all) explain your answer. Will you continue to vaccinate your son/daughter with the second dose if you have vaccinated him/her with the first dose? explain your answer; (3) Are you satisfied by the response you received from the Ministry of Health? 1 (very much) to 7 (very little). Please explain your answer whether you are very satisfied or not: (4) What do you feel after reading the health ministry's response? Please make a circle around the aspect that best describes your feelings on a scale of 1 (very much) to 7 (very little) out of the following questions: (4.1) Do you feel you have the tools to make a decision about sending your child to the kindergarten?, (4.2) Do the Ministry of Health’s tools help you make a decision about sending your child to the kindergarten?; (4.3) What do you think of the effectiveness of the Ministry of Health’s response?; (5) After the Ministry of Health’s response will you continue to look for information? (Yes/No). In light of that response, what do you want to know?; (6) In light of that response by the Ministry of health, who will you turn to for information about the measles vaccine? Please mark the main two sources you would turn to (list of sources); (7) Who do you think is the most reliable source of information after reading the Health Ministry's response? (Please mark a single answer); (8) Do you think the Ministry of Health’s answer is credible? (Yes/No); Please make a circle around the degree of credibility of the Ministry of Health's response on a scale of 1 (very much) to 7 (very little). Make a circle around your answer and explain it; (9) Have you been exposed to previous announcement by the Ministry of Health using similar language? Specify your answer. (Yes/No); (10) Did the Ministry of health influence your position? Please make a circle around the aspect that best describes your position on a scale of 1 (very much) to 7 (very little) out of the following statements: (10.1) Strengthen my position, (10.2) Weaken my position, and (10.3) Did not strengthen or weaken my position. explain your answer.

### Analysis

The study combined quantitative and qualitative research methods of data analysis.

#### Quantitative analysis

First distributions were tested for the demographic questions. Then the questions were tested on all subjects comparing Condition 1 to Condition 2 concerning Health Ministry reliability, level of satisfaction with the Health Ministry response, self-efficacy, further information seeking, and behavioral intentions regarding vaccination and sending children to kindergarten. Simultaneously an analysis was conducted comparing pro-vaccination and hesitant subjects on all of the aforesaid variables. An analysis and comparison were conducted between subjects who had children and those without children, so that each analysis was run 5 times: (1) whole sample; (2) for pro-vaccination; (3) for hesitant; (4) for respondents who had children and (5) for respondents without children. The analysis was conducted by the following tests:

1. A comparison between Condition 1 and Condition 2 on reliability of the Health Ministry’s response and satisfaction with the Health Ministry’s response. Both variables were tested after the response. The comparison was done using a t-test for independent samples.
2. A comparison between Condition 1 and Condition 2 as to further information searching. The variable was tested after the Health Ministry’s response. The comparison was made using a chi-square test.
3. The association between three levels of self-efficacy–low, medium and high–and further information searching was tested for Condition 1 and 2. The variable was tested after the Health Ministry’s response using a chi-square test. The objective of this analysis was to test an additional result of low self-efficacy, which is the need to seek for information.
4. A comparison in both Condition 1 and 2 of self-efficacy between the case description (Story 1) and the mother’s post (Story 2). The comparison was done using a t-test for dependent samples. This was done just to verify there was no change in self-efficacy until the manipulation of the experiment (story 3) appears.
5. A separate comparison to Condition 1 and Condition 2 of self-efficacy between the case description (Story 1) and the Health Ministry’s response (Story 3). The comparison was done using a t-test for dependent samples. This was done for the examination of the change in self-efficacy after the manipulation of the experiment (story 3) appears, compared to the baseline self-efficacy.
6. A separate comparison of Condition 1 and Condition 2 of self-efficacy between the mother’s post (Story 2) and the Health Ministry’s response (Story 3). The comparison was done using a t-test for dependent samples. This was done for the examination of the change in self-efficacy after the manipulation of the experiment (story 3) appears–compared to the self-efficacy after the mother’s post.
7. Examining the association in Condition 1 and Condition 2 between satisfaction with the Health Ministry’s response and behavioral intention to vaccinate child and behavioral intention to send child to kindergarten, for each experiment group separately using a logistic regression.

#### Qualitative analysis

The findings of the open questions were analyzed using content analysis [75]. In order to find recurring subjects, first the main themes and subthemes of each version of questionnaires (Conditions 1 and 2) were coded separately. In the second stage they were integrated, and groups of common themes were created by comparison between the two conditions.

### Reliability and validity test

We conducted content validity test in constructing the experiment in three stages. The first stage was constructing the experiment questionnaires. The construction of the questionnaire for Condition 1 used the tone and wording of common Health Ministry statements. The construction of the questionnaire for Condition 2 (theory-based) used the risk/health communication criteria [76] for writing on social networks [8, 77]. In the second stage we used face validity[78] and conducted a pilot with 10 people and tested the subjects' understanding of the wording while making corrections. The third stage was a pilot experiment with 47 subjects, and after we realized that the experiment was comprehensive we decided to expand it.

### Ethics

The study was approved by the Committee on Health and Welfare Sciences, The Faculty of Social Welfare and Health Sciences at the University of Haifa, confirmation number 421/17. All the study participants gave their written consent to participate in the research and publish its results.

## Results and discussion

The findings will be presented in three stages. In the first stage we will present the sociodemographic characteristic of the participants (see Table 1). In the second stage we will present the findings for all of the participants, backing them up with quotes from analysis of the open qualitative questions. In the third stage we will present the findings in relation to the subgroups: pro-vaccination and hesitant. We also ran tests divided by participants who have children compared to ones who do not have children, however no differences were found between them.

**Table 1:** Sociodemographic characteristics of the research participants.

Sociodemographic characteristics	Category	n (%)
**Sex**	Men	40 (16.5)
	Female	201 (82.7)
**Age (years)**	Mean (Max, Min, SD)	30.0 (54.0, 18.0, 7.71)
**Marital Status**	Single	122 (50.2)
	Married	108 (44.4)
	Divorced	7 (2.9)
	Widowed	2 (0.8)
**Number of children**	One	17 (7)
	Two	32 (13.2)
	Three	25 (10.3)
	Four	13 (5.3)
	Five	1 (1.2)
	None	150 (61.7)
**Ethnicity**	Jewish	153 (63.0)
	Arab	68 (28.0)
	Druze	13 (5.3)
	Other	5 (2.1)
**Education**	High school	27 (11.1)
	Post-high school	12 (4.9)
	BA	62 (25.5)
	MA	83 (34.2)
	PhD	4 (1.6)
	Other	8 (3.3)

### Findings for overall subjects

We began the comparison with a t-test between Condition 1 and Condition 2 (Table 2) on the variable of reliability of the Health Ministry’s response (“Do you find the response of the Health Ministry reliable?”), after the participants read the response, which was different for Condition 1 and Condition 2. A significant difference was found in the reliability level attributed to the Health Ministry's response between Condition 1 and Condition 2 (sig<0.001), with the average reliability level of the participants in Condition 2 (M = 5.68) being considerably higher than the average reliability level of the participants in Condition 1 (M = 4.64).

**Table 2:** Comparison between reliability and satisfaction of subjects between Condition 1 and Condition 2.

Variable	Condition 1			Condition 2			Significance (t test)
	n	Mean	SD	n	Mean	SD	
Reliability of Health Ministry’s response	118	4.64	1.77	110	5.68	1.39	<0.001
Satisfaction with Health Ministry’s response	122	4.66	1.83	114	5.75	1.24	<0.001

Subsequently we tested the difference on the variable of satisfaction with the Health Ministry’s response (“Are you satisfied by the response you received from the Health Ministry?”) A significant difference was found between Condition 1 and Condition 2 (sig<0.001), with the average satisfaction with the Health Ministry’s response of Condition 2 participants (M = 5.75) being significantly higher than the average satisfaction of Condition 1 subjects (M = 4.66).

This finding is also reinforced by the qualitative analysis. In focusing on the Health Ministry’s response, 43(34.1%) participants in Condition 1 noted that the organization’s response did not address concerns and did not provide scientific and sufficient information (79.6% out of participants who expressed their positions about the Health Ministry). For example:

“Most of the Health Ministry’s response is intimidation and causing parents anxiety without providing detailed information about the efficacy and benefits of the vaccination and side effects, if there are any."

In contrast, only 12(10.3%) participants in Condition 2 (25.5% out of participants who expressed their position about the Health Ministry) noted that the Health Ministry’s response was unsatisfactory and failed to address all of the parents' questions and concerns. The response of the Health Ministry in Condition 2 was described as a persuasive response by 43(36.8%) participants (91.4% out of participants who expressed their position about the Health Ministry). For example:

"I am pro-vaccination, I vaccinate my children and will continue to do so by the book. But the ministry’s position on this definitely reinforces my position towards vaccinations.”

#### Information searching

We tested further information searching following the Health Ministry’s response ("After receiving the Health Ministry’s response will you continue searching for information?”) by a chi-square test (Table 3). No significant difference was found between Condition 1 and Condition 2 as to further information searching after the Health Ministry’s response. Most participants continued to seek for information after the Health Ministry’s response both in Condition (81%) and Condition 2 (79%).

**Table 3:** Comparison of further information seeking following Health Ministry’s response between Condition 1 and Condition 2.

Variable	Condition 1		Condition 2		Significance (chi-square test)
	n	Yes (%)	n	Yes (%)	
Further information search after Health Ministry’s response	118	96 (81)	113	89 (79)	0.6215

This finding is also reinforced by the qualitative analysis. In both experiment groups many of the participants who were satisfied with the Health Ministry’s response, as well as many participants who were less satisfied, declared that they would seek additional sources of information. In Condition 1, 64(50.8%) participants noted in at least one story that they would seek further information, compared to 59(50.4%) participants in Condition 2 who noted that they would seek information. For example, one participant in Condition 1 noted that:

“I would like to know what it is for. What is its purpose? Pros and cons, to check what is preferable, are there more pros or cons? This is after I received true and correct information, not erroneous, partial or misleading information.”

In addition, two participants in Condition 2 noted that:

“I would like to know more about the frequency of the disease, risk of getting sick after the vaccination, side effects of the vaccination, complications of the vaccination compared with the sickness.”

“Scientific studies on the efficiency of the vaccination, its risks for the human body.”

We continued examining the questions of information searching and self-efficacy and divided efficacy into three levels: 1–2 low efficacy, 3–5 medium efficacy, and 6–7 high efficacy. We ran a chi-square test to analyze the differences between efficacy levels concerning information searching on the subject after reading the Health Ministry’s response (Table 4). We found significant gaps between the different levels of self-efficacy concerning information searching in Condition 1 (sig<0.05). At low levels of efficacy, 19(86%) participants continued searching for information, at medium levels of efficacy, 44(94%) participants continued searching for information, and at high levels of efficacy, 20(71%) participants continued searching for information. We can see that at medium levels of efficacy the number of participants who continue searching for information is the highest compared to participants with low or high efficacy. No statistically significant difference was found between efficacy levels and information searching in Condition 2, but even in Condition 2 group, the highest number(percentage) of participants who continued searching for information, were those with a medium level of self-efficacy.

**Table 4:** Self-efficacy and information searching in Condition 1 and Condition 2.

Variable	Self-efficacy						Significance (chi-square test)
	1–2		3–5		6–7		
Further information search after Health Ministry’s response	**n**	**Yes (%)**	**n**	**Yes (%)**	**n**	**Yes (%)**	
Condition 1	22	19 (86)	47	44 (94)	28	20 (71)	0.0301
Condition 2	20	15 (75)	32	28 (88)	39	29 (74)	0.3498

We pored deeper into the self-efficacy variable (“Do you feel you have the tools to make a decision about sending your child to kindergarten?”) and ran within-subjects t-tests (Table 5) that compared the feeling of self-efficacy of participants in Condition 1 and Condition 2 after the case description (Story 1), after the mother's post (Story 2), and after the Health Ministry’s response (Story 3). Stories 1 and 2 in both conditions were identical and the difference between them began only at the stage of the Health Ministry’s response (Story 3). No difference was found between the feeling of self-efficacy of participants after the case description (Story 1) compared to that feeling after the mother's post (Story 2), both in Condition 1 and Condition 2.

**Table 5:** Self-efficacy, within-subjects tests in Condition 1 Condition 2.

Story and comparison	Condition 1 (n = 63)		Condition 2 (n = 57)		All (n = 120)	
	Mean	SD	Mean	SD	Mean	SD
Story 1 self-efficacy	4.76	1.68	4.84	1.77	4.80	1.72
Story 2 self-efficacy	4.97	1.67	5.04	1.90	5.00	1.78
Story 3 self-efficacy	4.81	1.68	5.42	1.79	5.10	1.76
Story1 vs. Story2 Significance (t-test)					0.1668	
Story1 vs. Story3 Significance (t-test)	0.8196		0.0150			
Story2 vs. Story3 Significance (t-test)	0.4554		0.1283			

In examining the differences in self-efficacy between the case description (Story 1) and the Health Ministry’s response (Story 3) a significant difference was found in Condition 2 (sig<0.05). Condition 2 participants felt on average higher self-efficacy (M = 5.42) after the Health Ministry’s response compared to the feeling of self-efficacy they had at the beginning of the experiment, after reading the case description (M = 4.84). It is worth noting that no similar finding was found in Condition 1.

#### Within-subjects self-efficacy tests

**Fig 1:**
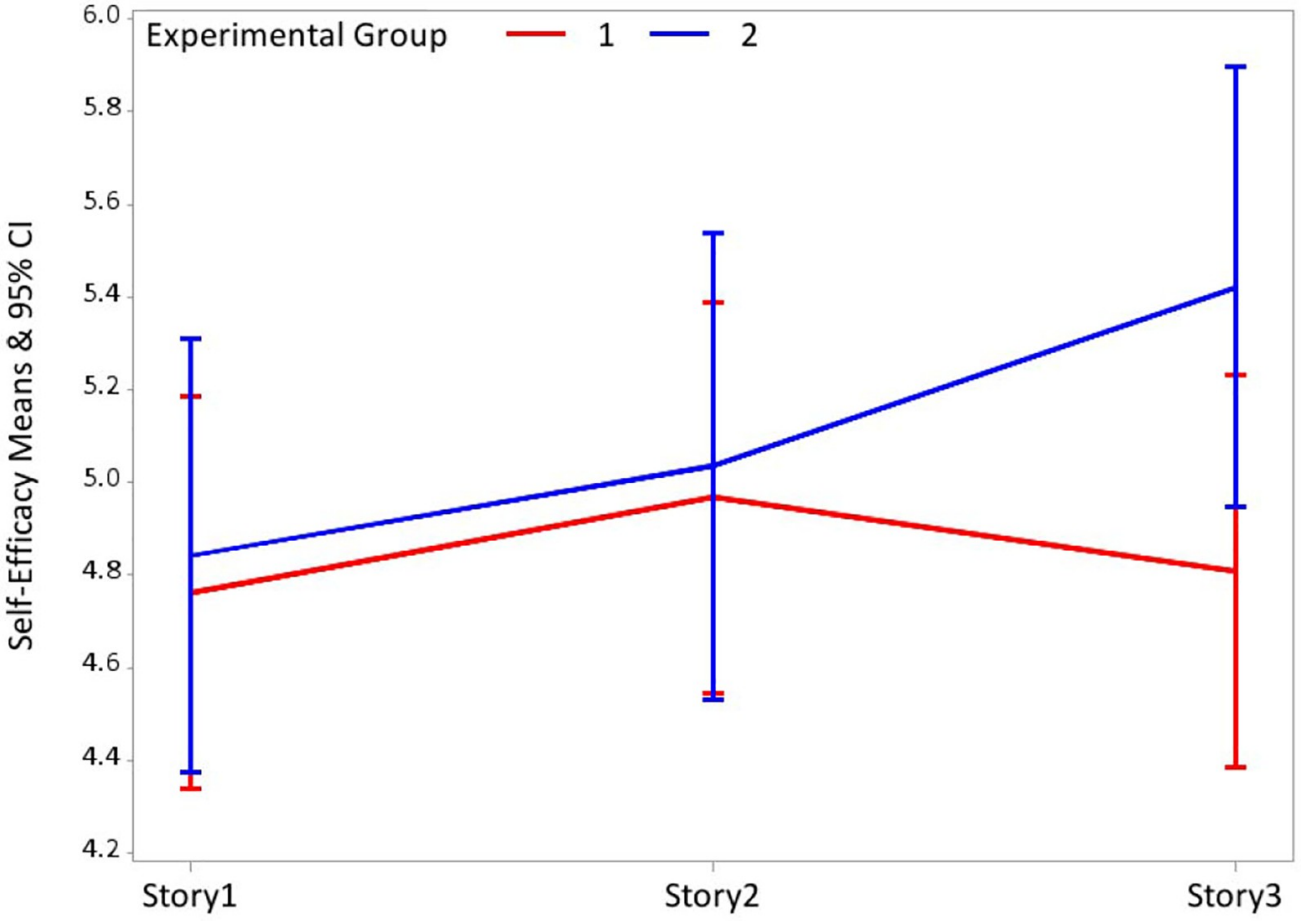
Participants' development in self-efficacy between the different stories.

Fig 1 shows the participants' development in self-efficacy between the different stories in the two conditions. There is a significant rise (sig<0.05) in feeling of self-efficacy in Condition 2 after the case description–Story 1 (M = 4.84) compared to that feeling after the Health Ministry’s response Story 3 (M = 5.42). Conversely, in Condition 1 no such significant rise in that feeling is observed after the case description–Story 1 (M = 4.76) compared to that feeling after the Health Ministry’s response Story 3 (M = 4.81).

We continued the tests and through a logistic regression we examined the association between satisfaction with the Health Ministry’s response and behavioral intention of the likelihood of sending a child to kindergarten (“After reading the Health Ministry's response will you send your son/daughter to kindergarten?”). We did not find a significant association between satisfaction with the Health Ministry’s response and behavioral intention to send child to kindergarten, neither in Condition 1 nor in Condition 2 (Table 6).

**Table 6:** Association between satisfaction and behavioral intention to send child to kindergarten.

Response Variable		Behavioral intention to send child to kindergarten				
Analysis of maximum likelihood estimates	Parameter	DF	Estimate	SE	Wald chi-square	Pr > ChiSq
Condition 1	Intercept	1	1.5103	0.5778	6.8329	0.0089
	Satisfaction	1	-0.1912	0.1111	2.9599	0.0854
Condition 2	Intercept	1	1.2974	1.1680	1.2338	0.2667
	Satisfaction	1	0.0622	0.2004	0.0963	0.7564

Using a similar logistic regression (Table 7) we examined the association between satisfaction with the Health Ministry’s response and behavioral intention to vaccinate child (“After reading the Health Ministry's response will you vaccinate your son/daughter?”). In Condition 1 a significant association was found between satisfaction and this behavioral intention. Thus, satisfaction increase the likelihood of sending the child to the kindergarten. The higher the participants' satisfaction with the Health Ministry’s response, the more they tended to vaccinate their children (sig<0.01).

**Table 7:** Association between satisfaction and behavioral intention to vaccinate child.

Response Variable		Behavioral intention to vaccinate child				
Analysis of maximum likelihood estimates	Parameter	DF	Estimate	SE	Wald chi-square	Pr > ChiSq
Condition 1	Intercept	1	-0.5005	0.7824	0.4092	0.5224
	Satisfaction	1	0.5464	0.1886	8.3938	0.0038
Condition 2	Intercept	1	-0.3833	1.4457	0.0703	0.7909
	Satisfaction	1	0.5217	0.2744	3.6156	0.0572

It should be noted that even though we would have expected to find a similar significant and positive association in Condition 2, no association was not found and this might be attributed to the fact that Condition 2 lacked statistical power for a regression because only seven of the Condition 2 participants said they would not vaccinate their children versus 80 who said they would. In such a case it is difficult to achieve enough power for a logistic regression.

### The hesitant group

Using a t-test we made a comparison of hesitants between Condition 1 and Condition 2 on the variable of reliability of the Health Ministry’s response, after the participants read the response, which was different for Condition 1 and Condition 2 (Table 8). A significant difference was found in the reliability level attributed to the Health Ministry’s response between Condition 1 and Condition 2 (sig<0.05), with the average reliability level of participants in Condition 2 (M = 5.11) being considerably higher than the average reliability level of participants in Condition 1 (4.17).

**Table 8:** Comparison between reliability and satisfaction of hesitant and pro-vaccination participants between Condition 1 and Condition 2.

Variables	Condition 1			Condition 2			Significance (t-test)
	N	Mean	SD	N	Mean	SD	
Hesitant							
Reliability of Health Ministry’s response	29	4.17	1.73	36	5.11	1.60	0.0269
Satisfaction with Health Ministry’s response	30	3.63	1.87	37	5.35	1.55	0.0001
Pro-vaccination							
Reliability of Health Ministry’s response	70	4.86	1.81	60	5.98	1.23	<0.0001
Satisfaction with Health Ministry’s response	72	4.94	1.76	62	5.97	1.04	<0.0001

We went on to check in a similar way the difference on the variable of satisfaction with the Health Ministry’s response among hesitants and found a significant difference between Condition 1 and Condition 2 (sig<0.001), with the average satisfaction of Condition 2 participants (M = 5.35) being significantly higher than the average satisfaction of Condition 1 participants (3.63).

### The pro-vaccination group

Using a t-test we made a comparison of pro-vaccination participants between Condition 1 and Condition 2 on the variable of reliability of the Health Ministry’s response, after the participants read the response, which was different for Condition 1 and Condition 2 (Table 8). A significant difference was found in the reliability level between Condition 1 and Condition 2 (sig<0.001), with the average reliability level of the participants in Condition 2 (M = 5.98) being considerably higher than the average reliability level of participants in Condition 1 (4.86).

Then we checked the difference on the variable of satisfaction with the Health Ministry’s response among pro-vaccination participates and found a significant difference between Condition 1 and Condition 2 (sig<0.001), with the average satisfaction of Condition 2 subjects (M = 5.97).

### Discussion

New media health communication is a great challenge for health organizations worldwide. The issue of how to implement effective activities to correct information on social media is at the center of the discussion in the literature [1, 73]. This study sought to examine participants’ different responses to the correction of misinformation by constructing an experiment that simulates a Health Ministry’s response on social networks. With new media online social networks have become the public sphere [1, 2]. The public conducts a lively discussion on social networks, exchanging views, providing information and discussing health issues, with the discussion of vaccinations being particularly lively [19, 20, 79]. In this study we ran an experiment in which we put the participants through different simulations in which we presented the dilemma of sending a child to a kindergarten where not all the children are vaccinated against the measles, a mother’s post, and a response of a Health Ministry to the post. Some of the subjects received a common response (i.e., the tone and the wording that can be found on Health Ministries' websites), versus a response based on the theory of health and risk communication. The findings of the study indicated that the experiment participants who received a Health Ministry response in keeping with the theory, expressed a higher level of satisfaction, trust and self-efficacy than those who received a common Health Ministry response. These findings support the assumptions of the approach of risk communication, namely that when health organizations provide full and transparent information and address the emotional element, they are more effective than when they deliver one-dimensional, partial responses that do not address the public's fears and concerns [8, 53, 77, 80].

Furthermore, the same result was found when we examined participants who were pro-vaccination. Which is to say that even the pro-vaccination group prefers to receive full information that addresses emotions and concerns and expresses less satisfaction with a response that does not address those aspects. This is an important finding, which might have future implications: it is possible that participants who maintain pro-vaccination positions and are not happy with the responses of the Health Ministry might in certain situations in the future become hesitant. Similarly, when we examined the hesitant group in the experiment we found again that the participants who received a theory-based response from the Health Ministry showed higher satisfaction than members in the experiment group who did not receive such a response. This indicates that a credible response by the health organization can increase the feeling of reliability and satisfaction of hesitants and this provides an opening for two-way communication between the hesitant group and the organizations.

The study also found that in the two conditions of the experiment, participants reported that they would continue searching for information even after the Health Ministry’s response to a post on the social network. This indicates that the organizations' conversation on social networks cannot be limited to “Q&A” or a time-limited dialogue, but as the convergence communication approach [33] indicates, it must be a circular and continuous dialogue that continues over time until convergence. The public wants to receive information about different aspects while addressing new questions that come up and is not satisfied with a single response, no matter how detailed.

Another finding of this study is that the highest percentage of participants who will continue to search for information are people with medium levels of self-efficacy in comparison with people with low or high self-efficacy. This might be explained by the fact that people with high levels of self-efficacy feel they have the tools to decide whether to send their child to kindergarten, whereas those with low levels of efficacy feel they do not have the tools and do not know how to search for information. Whereas those who are in the middle on the one hand do not have all of the tools and on the other hand do have the skills and reasonable literacy to find information, so they continue to search.

Research limitations are that this was not a representative sample of the whole pro-vaccination and hesitant publics, but from the beginning as an experiment design it was not intended to be. Furthermore, since the participants included both pro-vaccination and hesitant groups, it was found that even among healthcare workers who work within the health system, there are some who define themselves as hesitant, and it is necessary to test even with this group how to correct misinformation rather than assuming that they are "a captive audience" of the system as regards to routine vaccinations for children. We must also remember that it is possible that among those who participated in the experiment there were parents who did not remember what happened when their children went to kindergarten. Furthermore, the parents participating in the simulation might respond differently than they did years ago when they had to make the decision. But this research does not intend to examining historical reliability. Rather, it examines current attitudes and behavior towards vaccinations. Furthermore, this study did not examine the anti-vaccination population (their number in this study was negligible). Another limit is that the study does not address whether the changes observed would be maintained over time. Follow-up studies should be conducted with those three subgroups, as well as follow-up studies to examine reactions in different time frames.

## Conclusions

The findings indicate that it is very important for the organizations to correct misinformation transparently while addressing the emotional aspect both for the pro-vaccination and the hesitant group. The fact that the pro-vaccination subjects were less satisfied with the "common" response indicates that the organizations must not treat the pro-vaccination public as a captive audience. In addition, the hesitant group was satisfied with the organization's response when it presented full information, which addressed fears and concerns, strengthening the assumption that health organizations can increase their reliability even vis-à-vis the hesitant group.
